# The prognostic significance of lymph node size in node-positive colon cancer

**DOI:** 10.1371/journal.pone.0201072

**Published:** 2018-08-10

**Authors:** Philipp Schrembs, Benedikt Martin, Matthias Anthuber, Gerhard Schenkirsch, Bruno Märkl

**Affiliations:** 1 Institute of Pathology, Klinikum Augsburg, Augsburg, Germany; 2 Department of Visceral Surgery, Klinikum Augsburg, Augsburg, Germany; 3 Clinical and Population-Based Cancer Registry, Augsburg, Germany; University of South Alabama Mitchell Cancer Institute, UNITED STATES

## Abstract

**Objectives:**

To (i) show the outcome benefits of enlarged lymph nodes in node-positive colon cancer cases, as it was shown previously in negative node cases; (ii) disprove the stage migration theory and (iii) list the factors affecting lymph node size and yield.

**Methods:**

A retrospective study including 234 node-positive colon cancer cases was scheduled and performed. All recovered lymph nodes (6969) from 234 cases were microscopically examined in regard to (a) lymph node size (b) presence of metastasis (c) extent of intra-nodal metastasis. On the basis of resulting data, a statistical analysis was performed.

**Results:**

Metastases occurred in all size categories, though more often in larger lymph nodes. Fifty-one percent of all metastasised nodes were 2 to 6 mm in size. Approximately half of all nodes >10 mm were microscopically free of cancer. Cases with a small *lymph node metastasis to lymph node size ratio* (MSR) had a better prognosis than others: 85 months (95% CI: 72–97) vs. 67 months (95% CI: 47–88), p <0.001 (mean, overall survival). To differentiate between cases with the same ratio but different absolute lymph nodes sizes, we divided the cases into two groups that differed in their number of moderate to large lymph nodes. The group with more moderate to large lymph nodes showed a clear outcome benefit: 104 months (95% CI: 86–122) vs. 66 months (95% CI: 54–77), p = 0.014 (mean, overall survival).

**Conclusions:**

Metastasised lymph nodes affect all size categories, and large lymph nodes are not always metastasised. The combination of enlarged lymph nodes and a small *lymph node metastasis to lymph node size ratio* (MSR) is associated with a better prognosis than others. When enlarged lymph nodes were considered as surrogate markers of an effective local immune response due to nodal hyperplasia, the immune system could be seen as the confounder affecting both lymph node size and prognosis. Our results are pointing in this direction and, along with other reasons, are challenging the stage migration theory.

## Introduction

In colon cancer cases, postoperative lymph node staging is of crucial importance for prognostic stratification and therapeutic sequelae. Still, surgery is the therapy of choice. Without evidence of metastasised lymph nodes or distant metastases, no further treatment is recommended. However, there are some well-defined risk factors that can determine the implementation of adjuvant chemotherapy in such cases [1]. A small number—fewer than 12—of postoperatively examined lymph nodes is one of those. The reason to allocate such cases to a risk group was that higher numbers of lymph nodes have been observed to be associated with better survival [2, 3]. Stage-migration theory was the most likely explanation for this phenomenon; low numbers imply the risk of missing metastasised lymph nodes, whereas plenty of lymph nodes ensure an adequate staging. However, since doubts have risen regarding the stage-migration theory, a better explanation is being sought. The awareness of the prognostic association as well as the launch of new and more effective lymph node dissection techniques in the context of quality initiatives in pathology institutes together led to a better mean lymph node yield per case of colon cancer [4]. However, despite significant lymph node yield improvements, no increase of node positivity rate could be noted [5, 6]. This is not consistent with the stage-migration model.

Therefore, some authors have pointed out that the stage-migration theory might be incorrect. To give an alternative explanation, the immune system was suggested as the confounder influencing the lymph node yield and survival and, therefore, the true explanation of outcome benefits.

Lymph node size is certainly associated with the number of examined lymph nodes because larger nodes are easier to find. Therefore, nodal size is suspected to be a semi-quantitative parameter of local immune response that connects better lymph node yield with survival benefits.

The prognostic relevance of nodal size is attracting increasing attention because promising results have been published in the past few years [7, 8].

## Materials and methods

### Case collective

A retrospective observational study with 266 node-positive colon cancer cases from 2002 to 2004 and 2007 to 2013 was scheduled and performed. The inclusion criterion was node-positive colon cancer treated with primary surgery with curative intent. Exclusion criteria were neoadjuvant radiochemotherapy, emergency resection, rectal cancer location, neuroendocrine tumours, syn- or metachronous tumours of the colon, and R1/2 resection. For survival analyses, a minimum follow-up time of two months was stated. Eventually, 234 cases remained for statistical analysis.

The period between 2005 and 2007 was not allowed to take part in the study because, during this time frame, the stepwise implementation of new lymph-node dissection techniques took place at the Pathology Institute of the Klinikum in Augsburg, Germany.

Follow-up data were provided by the Clinical and Population-Based Cancer Registry of Augsburg. This study was approved by the Internal Review Board of the Klinikum, Augsburg, Germany.

### Lymph node dissection and parameters

Lymph nodes of cases from 2002 to 2004 were dissected conventionally; those from 2007 to 2013 were dissected mainly by advanced techniques such as the methylene blue–assisted lymph node dissection technique (MBLND). This technique has been described before [9].

Using a digital camera and appropriate software (ProgRes C10 and C3, Jenoptik, Jena, Germany), all surgically salvaged lymph nodes were examined microscopically (hematoxylin and eosin [H&E]-stained slides) with regard to size, the presence of tumour infiltrate and, if present, size of tumour infiltrate. The lymph node sizes were categorised according to their maximum diameter within 11 groups. The first category comprised nodes ≤ 1 mm; the second category comprised those ≤ 2 mm and so on. The 11th group consisted of nodes > 10 mm. In addition, a l*ymph node metastasis to lymph node size ratio* (MSR) was calculated for each case. This value bears subtle information about the extent of disease in the lymph nodes and is calculated as follows: $\frac{\sum_{i=1}^{m}x_{i}}{\sum_{k=1}^{n}y_{k}}$ (x_i_ = max. diameter of tumour infiltrate in mm; m = total number of metastasised nodes per case; y_k_ = max. diameter of lymph node in mm; n = total number of lymph nodes per case). A ratio of 1.0 equals a complete masking of healthy lymph node tissue by tumour infiltrate. Cases with MSR < 0.1070 were designated as group 1, those with MSR > 0.1070 as group 0. Moreover, we applied the LN5 classification to give a measure of the number of moderate to large lymph nodes (> 5 mm) per case: LN5(+) ≥ 7 nodes > 5 mm, LN5(–) < 7 nodes > 5mm. The latter was introduced in former articles [8].

### Mismatch-repair enzyme status

Retroactively for all cases, fresh paraffin sections were prepared and antibody-stained for immunohistochemical determination of MMR (mismatch-repair)-enzyme status. The following diagnostic antibodies were used: PMS2 (Clone EP51, ready to use) and MSH6 (Clone EP49, ready to use). All reactions were developed using the Ventana Ultravision detection system (Roche Diagnostics, Mannheim, Germany).

### Statistics

Linear regression analysis was applied to test the association between two characteristics. For normally distributed and continuous data, Student’s *t*-test was used; when not normally distributed, we switched to the Mann-Whitney rank sum test. By means of the χ^2^ test, we analysed binary characteristics. The mean follow-up period was calculated with the method from Schemper and Smith [10]. Cut-off values were calculated with ROC (receiver operating characteristic) curves. We employed the log-rank test for comparison of overall survival times, including censored data. To visualise the results of the latter, Kaplan-Meier curves were plotted. For multivariable analysis, the Cox regression method was used. P-values < 0.05 were considered significant, and 95% confidence intervals (CI) were given where it was possible. Mean values are given with ±1 standard deviation (SD). All computations were made with SigmaPlot software 13.0 (Systat, Richmond, VA, USA).

## Results

### Clinicopathological data

Clinicopathological data are given in Table 1. There were 234 node positive colon cancer cases for analysis. The mean age was 68.5 ± 13, and 53% were male. With a mean follow-up period of 70 months (median: 65), the overall mortality rate was 54%. The mean number of examined lymph nodes was 30 ± 18. The average had 4 ± 6 positive nodes. In total, 31 cases had fewer than 12 examined lymph nodes (= insufficient pN-status).

**Table 1:** 

	Case collective n = 234	MSR group 0, n = 121	MSR group 1, n = 113	p-value	MSR group 1 with LN5(+) n = 57	MSR group 1 with LN5(-) n = 56	p-value
Gender, f:m	0.88:1	0.89:1	0.88:1	0.921	1.11:1	0.70:1	0.297
Age, mean ± SD	68.5 ± 13	68 ± 13	69 ± 13	0.330	67 ± 15	70 ± 11	0.438
Insufficient pN-status	31	26	5	<0.001	0	5	0.064
**Examined LN per case, mean ± SD**	30 ± 18	25 ± 16	34 ± 18	<0.001	40 ± 19	28 ± 16	<0.001
**Positive LN per case, mean ± SD**	4 ± 6	6.68 ± 7.83	1.80 ± 1.14	<0.001	1.96 ± 1.20	1.62 ± 1.0	0.080
Common adenocarcinoma	191	91	100		49	51	
Other histology	43	30	13	0.014	8	5	0.578
pT1/2: pT3/4	23/211	7/114	16/97	0.054	4/53	12/44	0.054
pN1: pN2	152/82	51/70	101/12	<0.001	51/6	50/6	0.785
Low grade: high grade	128/106	59/62	69/44	0.079	30/27	39/17	0.079
Distant metastases, M1:M0	71/163	72/49	91/22	<0.001	10/47	12/44	0.777
Right hemikolon	103	49	51		31	20	
Left hemikolon	104	48	51	0.945	20	31	0.048
MMR proficient	195	103	92		45	47	
MMR deficient	30	11	19	0.147	10	9	0.966
Adjuvant chemotherapy	147	79	68	0.236	35	33	0.937

147 out of 243 node positive patients received chemotherapy with certainty. In 23 cases there were no clinical data concerning available. Still there remain at least 64 patients who did not undergo chemotherapy either due to refusal of the patients or contraindications.

Within MSR group 0, it was more likely to encounter insufficient lymph node numbers (26 vs. 5), the average number of examined lymph nodes per case was smaller (25 vs. 34) and there were considerably more positive nodes per case (6.7 vs. 1.8) than in group 1.

After the dichotomous splitting of the 113 MSR group-1-cases into LN5(+) and LN5(–), the imbalance of the number of examined lymph nodes remained mainly apparent.

### Lymph nodes

**Fig 1:**
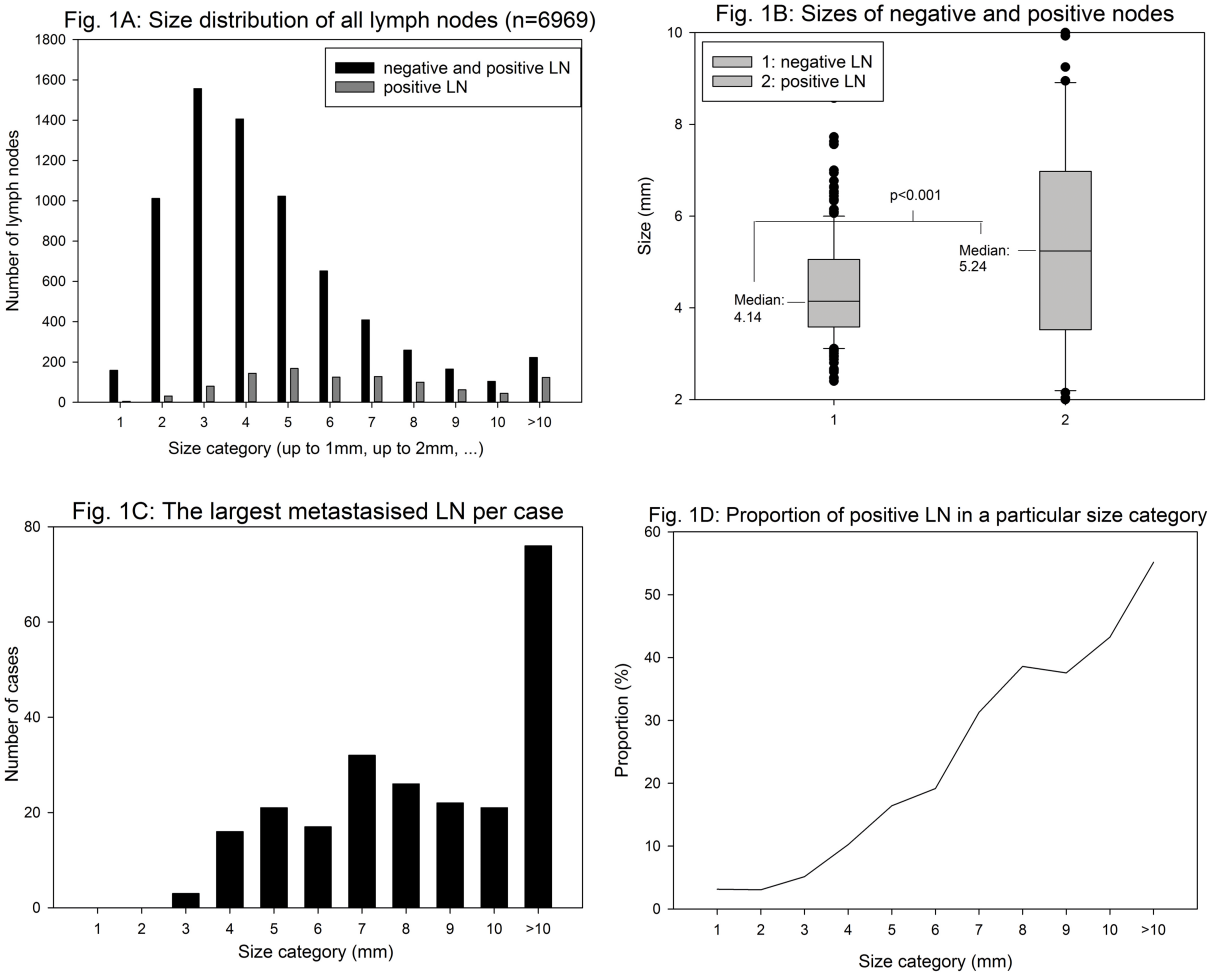
Size distribution of all lymph nodes (A); difference of size of negative and positive nodes (B); largest metastasised lymph node per case (C); percentage of positive lymph nodes in a particular size category (D). LN: lymph node.

In total, 6969 lymph nodes were microscopically investigated, and 1011 of them were metastasised (14.5%). The majority of lymph nodes were > 1 and ≤ 6 mm in size (Fig 1A). From all metastasised nodes, 51% measured between 2 and 6 mm. Forty-five percent of the lymph nodes > 10 mm were microscopically free of tumour cells. The mean sizes of positive and negative nodes were 5.7 ± 3.4 (median: 5.2) and 4.4 ± 1.1mm (median: 4.1), respectively (p < 0.001) (Fig 1B).

There were only four cases with positive nodes < 1 mm. Furthermore, in none of the cases the largest positive node was < 2 mm. Although small lymph node metastases indeed occurred, in most cases there were also considerably larger lymph node metastases to identify (Fig 1C). Fig 1D shows the percentage of positive nodes as a function of size category; the portion of positive nodes increases almost monotonously with size category (Fig 1D).

### Determining factors of lymph node number

Lymph node size, MMR-enzyme status, patient’s age, tumour localisation and T-stage were investigated regarding their impact on the number of evaluated lymph nodes.

After analysis, only lymph node size [LN5(+)/(–): 32.8 ± 19.6 vs. 25.7 ± 14.7 lymph nodes, p = 0.007] and MMR-enzyme status (MMR-deficient/proficient: 36.6 ± 18.8 vs. 29.2 ± 17.6 lymph nodes, p = 0.017) were found to be associated with the number of evaluated lymph nodes. It is worth mentioning that the nodal size was assessed as the number of nodes > 5 mm (LN5 classification).

### Determining factors of lymph node size

MMR-enzyme status, tumour localisation, T-stage and nodal status were investigated regarding their impact on the lymph node size (number of nodes > 5 mm).

MMR-enzyme status (MMR-deficient/proficient: 10.1 ± 7.4 vs. 7.3 ± 5.5, p = 0.045), tumour localisation (right and left hemicolon: 9.6 ± 6.1 vs. 5.9 ± 4.7, p < 0.001) and nodal positivity (metastasised/non-metastasised node: 5.7 ± 3.4 vs. 4.4 ± 1.1 mm, p < 0.001) were associated with the lymph node size.

### Survival analysis

The following variables were investigated regarding their survival predicting value: the number of evaluated lymph nodes, LN5 status and MSR.

**Fig 2:**
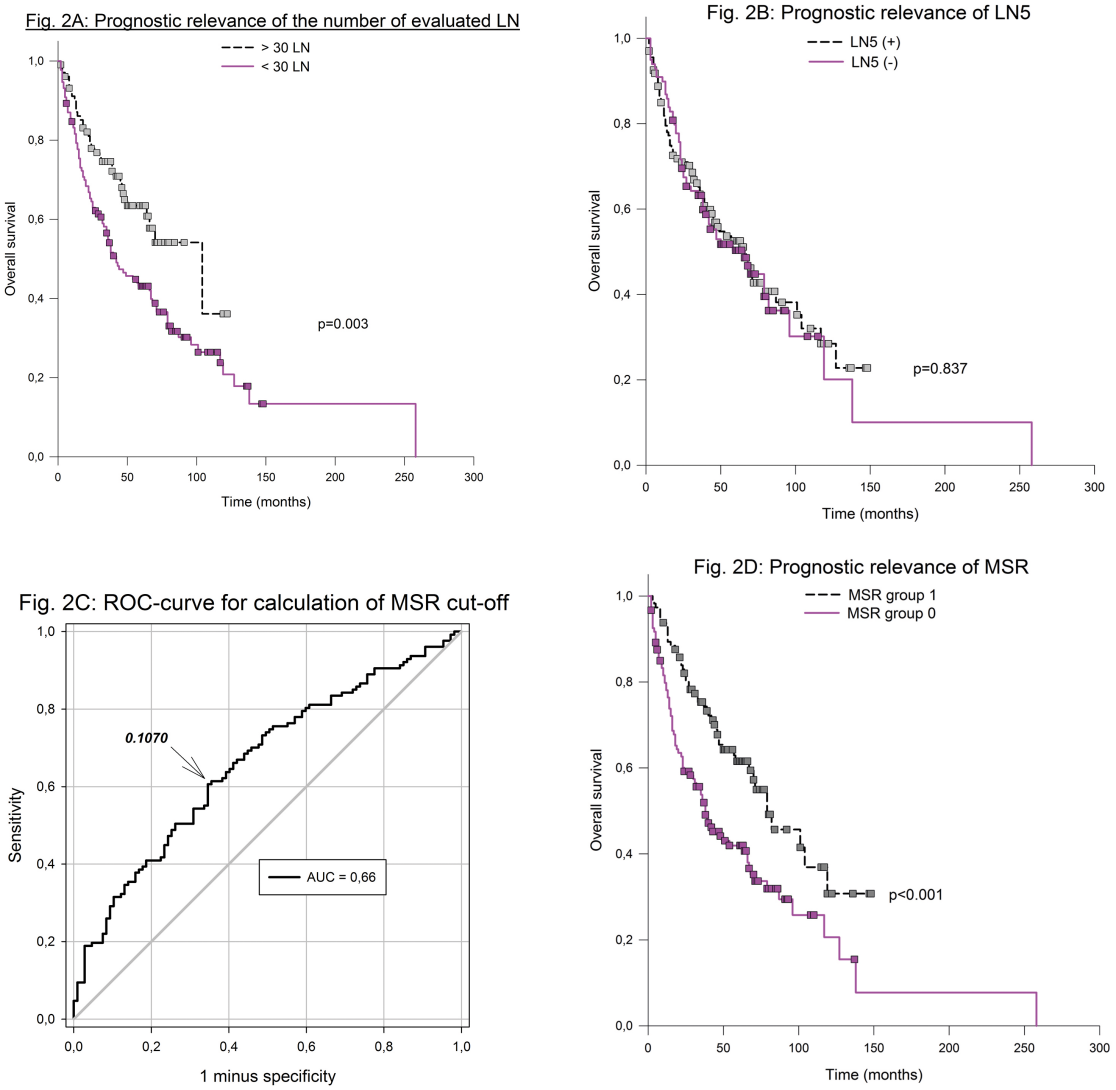
Prognostic relevance of the number of evaluated lymph nodes (A); prognostic relevance of LN5 alone (B); ROC curve for calculation of MSR cut-off (C); prognostic relevance of MSR (D).

Cases with more than 30 evaluated lymph nodes per case were associated with a better outcome, with median overall survival of 104 months, 95% CI: 51–157 vs. 42 months, 95% CI: 28–56, p = 0.003 (Fig 2A).

LN5 classification alone was not prognostic, with median overall survival times of 66 months, 95% CI: 49–83 (LN5[+]) vs. 66 months, 95% CI: 39–93 (LN5[–]), p = 0.837, respectively (Fig 2B).

However, the MSR was prognostically relevant. The ratio cut-off (0.1070) was calculated with an ROC curve (Fig 2C). Patients with a ratio < 0.1070 (MSR group 1) considerably differed from those with larger ratios (MSR group 0) through a longer overall survival: a median overall survival of 79 months, 95% CI: 54–103 vs. 38 months, 95% CI: 25–50, p < 0.001 (Fig 2D).

**Fig 3:**
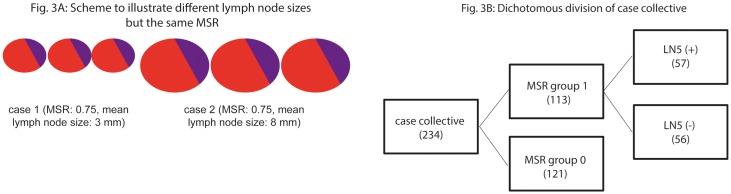
Virtual scheme to illustrate different lymph node sizes but the same MSR (A); dichotomous division of case collective (B).

**Fig 4:**
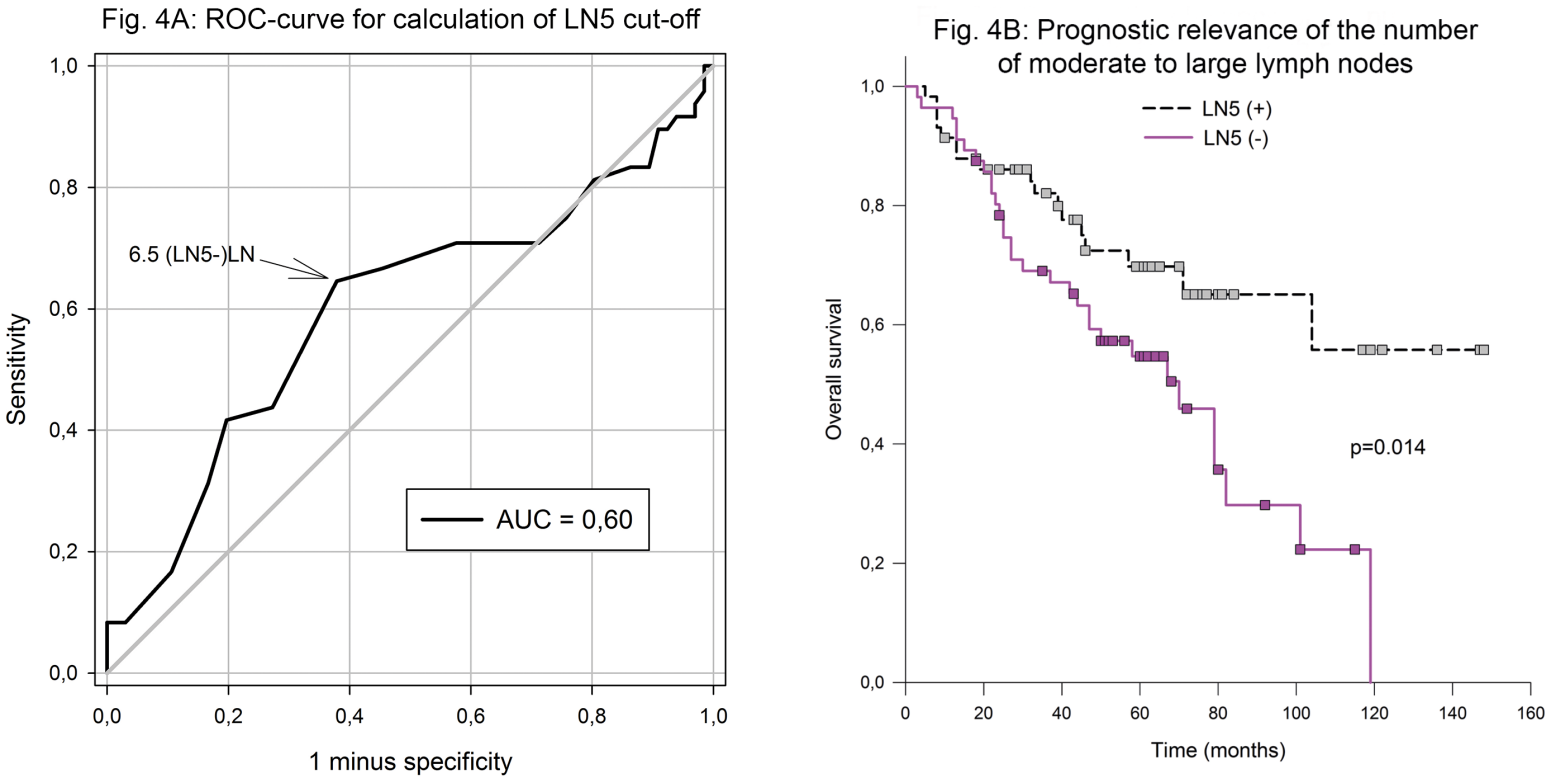
Prognostic relevance of the number of moderate to large lymph nodes (LN5) in those cases with MSR < 0.1070 (A); ROC curve for calculation of LN5 cut-off (B). LN: lymph node; LN5(+/–): ≥ 7/< 7 lymph nodes > 5 mm; MSR: lymph node metastasis to lymph node size ratio; AUC: area under the curve.

Even though the MSR had already integrated the sizes of individual lymph nodes (see Materials and methods), it was still necessary to employ a further factor to differentiate cases with the same ratio but different lymph node sizes (Fig 3A). Therefore, the LN5 classification was applied to MSR group 1. After dividing the 113 cases of MSR group 1, 57 LN5(+) cases and 56 LN5(–) cases (Fig 3B) were left. The former showed a clear survival benefit compared to the latter: mean overall survival 104 months, 95% CI: 86–122 vs. 66 months, 95% CI: 54–77, p = 0.014 (Fig 4A and 4B). The median of LN5(–) was 70 months; however, LN5(+) did not reach the median; hence, the mean values are given.

### Multivariable analysis

For the purpose of selection of those variables, which should be respected in the multivariable model, univariable analyses were made in advance. Distant metastases were excluded from this on purpose, because the prognostic power of these is so large that other factors would have been outshone if distant metastases had found allowance for multivariable testing.

Prognostically relevant were insufficient pN-status (< 12 nodes examined), number of evaluated nodes, T- and N-stage and tumour grading. The MMR status just missed significance with a p-value of 0.081. Adjuvant chemotherapy clearly missed statistical significance (p = 0.665).

After multivariable analysis with means of Cox regression MSR alone (p = 0.028, HR: 4.047), MSR with LN5(+) (p = 0.005, HR: 2.440), T-stage (p = 0.016, HR: 2.506), N-stage (p = 0.002, HR: 1.940) and grading (p = 0.003, HR: 1.739) were identified to be directly and independently associated with the patient’s overall survival.

## Discussion

In colon cancer diagnosis, lymph node staging is essential and bears important implications for further treatment; a preoperative N-staging can be differentiated from a postoperative N-staging.

Imaging methods rely—besides others—on lymph node size (> 10 mm) to estimate the probability of lymph node metastases, but then several studies report the inadequacy of the nodal size as a single marker to predict nodal involvement [11, 12]. Indeed, in our study, metastasised nodes were on average somewhat larger than non-metastasised (5.7 ± 3.4 vs. 4.4 ± 1.1 mm). Compared to previous results of a study with only node negative cases the overall size distribution of lymph nodes did not differ significantly [8]. Concerning this we cannot think that metastatic cancer cells in lymph nodes imply the enlargement of lymph nodes in general. Even though the probability of nodal involvement increased with size category (Fig 1D), 51% of all metastasised nodes measured between 2 and 6 mm, and 45% of nodes > 10 mm were (microscopically) free of tumour cells. Märkl et al. and Rössler et al. even stated that 72% and 74% of nodes > 10 mm, respectively, were free of tumour cells [8, 13]. These facts cause concerns regarding the diagnostic potential of imaging methods in preoperative lymph node staging in colon cancer cases.

A higher number of evaluated lymph nodes per case are associated with a better prognosis. This number–prognosis relationship has been tested several times and has been confirmed again in most studies [2, 3, 14]. First, the so-called stage migration effect was designated to be the underlying reason; low numbers imply the risk of missing metastasised lymph nodes, whereas plenty of lymph nodes ensure an adequate staging, but important observations gave a reason to question the stage migration theory.

Although the number of average evaluated lymph nodes has risen in the past decades through better surgical standards and introduction of new lymph node dissection techniques (e.g., MBLND), the rate of node-positive colon cancer, surprisingly, did *not* increase [5, 6, 15–17]. Independent from advanced dissection techniques, 57 studies from 1987 to 2015 were analysed with regard to the chronological development of the rate of nodal positivity [14]. The mean rate was 39%. Interestingly, the rate of inadequate lymph node harvest (< 12 lymph nodes) had no influence on the rate of node positivity [14]. One would have expected that the node positivity rate would decrease as the rate of inadequate lymph node harvest increased, so a better part of metastasised nodes was detected even if the nodal harvest was inadequate. The results of Goldstein et al. point in the same direction [4].

These are just a few observations challenging the stage migration theory. We addressed this topic in a systematic review recently [14]. As a consequence, the stage migration theory is hard to sustain, and a new, better explanation for the number–prognosis relationship must be found.

**Fig 5:**
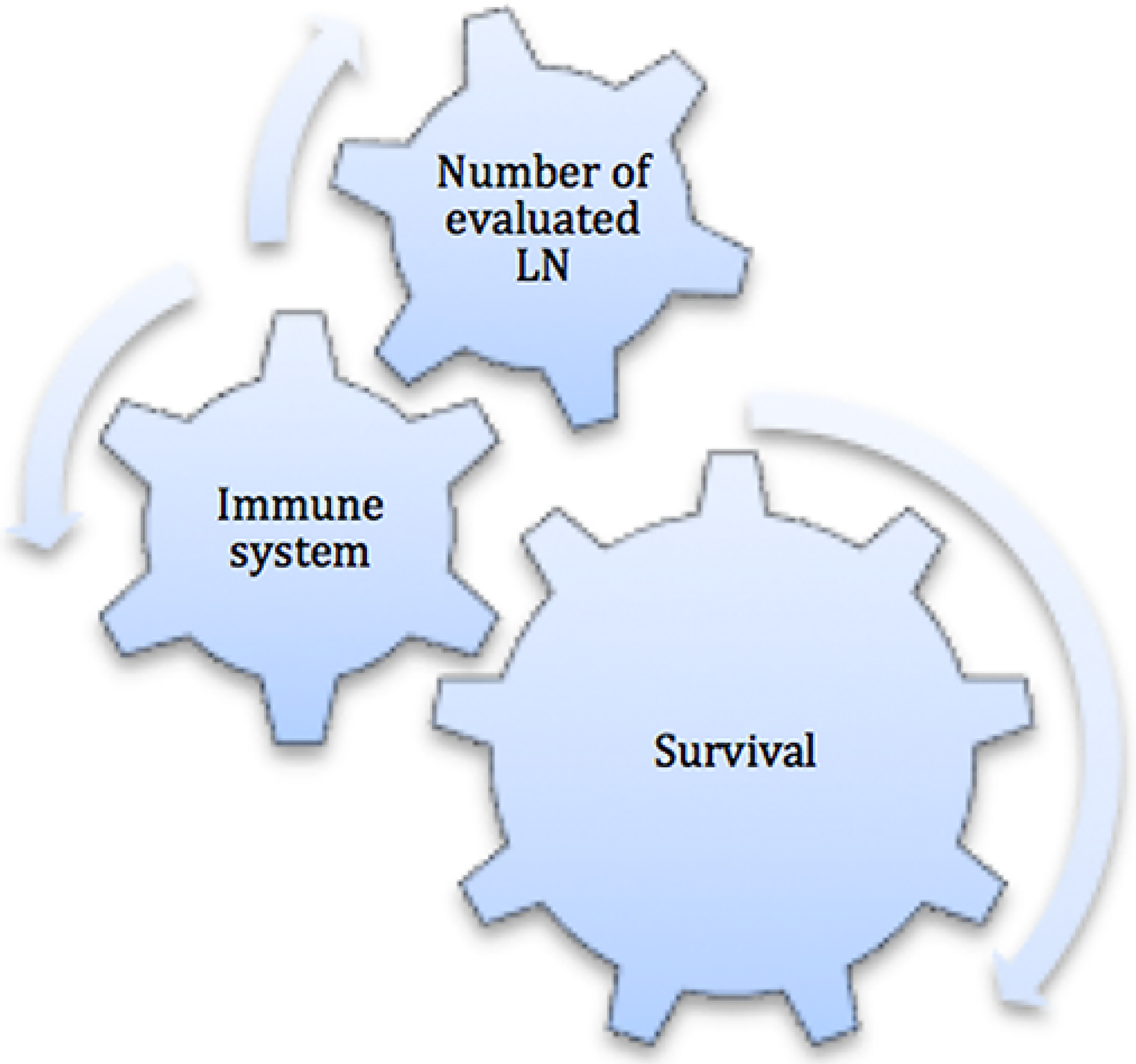
Immune system as confounder influencing both number of evaluated LN and survival. LN: lymph node.

The immunology model could be a reasonable explanation. For this purpose, the immune system assumes the key role of a confounding factor. A confounder is something that affects both an influencing factor and a dependent variable and, therefore, leads to wrong conclusions. The immune system influences both the number of evaluated lymph nodes and the prognosis (Fig 5). A strong local antitumour lymphocytic reaction causes hyperplastic enlargement of regional lymph nodes, making them easier to find during dissection in pathology laboratories [18–20]. Hence, we examined the connection between moderate to large lymph nodes and patients’ survival.

Former studies have already shown that indeed this connection is reasonable. For this purpose, Märkl et al. and Mayr et al. analysed 237 N(+)/(–) and 115 N(–) colon cancer cases, respectively, and could point out the positive correlation between the number of LN5 lymph nodes and survival [7, 8]. The number of LN5 nodes was a measure of how many moderate to large lymph nodes were present per case.

The application of the same method (LN5) to our exclusive node-positive case collection did not reach the statistical 0.05 level (Fig 2B). To correct for the bias of highly enlarged lymph nodes due to bulging metastasis, the MSR was introduced, because those lymph nodes should not be counted as immune-mediated enlarged nodes. MSR resembles the ratio of metastatic to resected lymph nodes (LNR), which has often proved to be an independent prognostic factor [21–24]; LNR divides the number of metastasized lymph nodes by the total number of evaluated lymph nodes. But considering the increase of lymph node yield with time (over the last decades there was a continuous increase) the total number of evaluated lymph nodes per case will rise and LNR consequently will drop. Smaller LNR predicts better prognosis. However, a plenty of very small lymph nodes as a consequence of enhanced dissection techniques will hardly improve patient’s prognosis. In contrast the MSR integrates individual lymph node sizes. Beyond that it also integrates the intra-nodal infiltrate size if present. For sure it is a more suitable parameter in the context of nodal size. In our opinion it is to early to estimate the chance to introduce MSR into clinical practice. MSR helped us to get rid of misleadingly enlarged lymph nodes and is a further hint that lymph node size plays an important role as a surrogate marker for immune response.

In our case collective MSR was an independent prognostic factor, too. Patients with a ratio < 0.1070 (MSR group 1) considerably differed from those with larger ratios through a longer overall survival: 79 vs. 38 months (median), p < 0.001. The prognostic independency was deduced from multivariable analysis.

Even though the MSR had already integrated the sizes of individual lymph nodes, it was still necessary to employ a factor to differentiate cases with the same ratio but different lymph node sizes. Therefore, the LN5 classification was now applied to the MSR group 1. In this subgroup, patients who tended to have more moderate to large lymph nodes could profit by a better survival: 104 vs. 66 months (mean), p = 0.014.

Rössler et al. investigated primary tumour characteristics affecting the lymph node size. High lymphocytic antitumour reaction had the highest hazard ratio among the others, but just missed statistical significance in the multivariable model (p = 0.053). In view of the statistical marginality, we can assume that this correlation is real and perhaps failed clear statistical significance due to a limited sample size (n = 148)[13].

In a recent study, we provided explicit clues for the interplay of the immune system with lymph node size and prognosis. We reconciled intra-tumoural T-lymphocytes (ITL) with high quantities of evaluated lymph nodes, larger lymph nodes and better prognosis [18]. Beyond that in another article the author’s group was able to show a positive correlation between the density of ITLs, patient’s survival, and number of enlarged lymph nodes (LN5) in node negative cases [18].

So did Kim et al. In their case collective, a strong inflammatory tumour infiltrate, quantified by means of conventional histology and immunohistochemistry, was a positive predictor for plenty of evaluated lymph nodes. In addition, among patients with stage III disease, a sparse inflammatory infiltrate was associated with worse survival. They reasoned that the volume of lymph nodes and the prognosis were affected by the antitumour immune response [20].

The high importance of tumour infiltrating lymphocytes has been shown in many studies by Galon et al. [25, 26].

Together, these facts confirm the immunology model.

### The role of small lymph nodes

Thirty-nine percent of all examined nodes were at most 3 mm in size. The proportion of positive nodes in the lower size categories was very small: 3% in size category 1, 3% in size category 2, 5% in size category 3 (Fig 1D). Although positive nodes occurred in all size categories, very small metastasised nodes have never been the only positive nodes found per case. In no single case, the largest positive lymph node found was < 2 mm in size (Fig 1C). Thus the very small metastasised nodes contribute to a precise N sub-classification (N1a/N1b/N2a, etc.) rather than to identify nodal positivity as such [8, 13]. Therefore, some authors raised the question of whether to search for the very small nodes at all [13, 14]. Currently, fewer than 12 examined lymph nodes represent a risk factor with subsequent implementation of adjuvant chemotherapy. Based on this and previous studies, we are convinced that the immune response plays a major role in the outcome of colon cancer. At least in part, insufficient lymph node yields might be a surrogate parameter for an impaired immune response. As the diagnostic techniques continuously progress (new lymph node dissection techniques, considerably higher numbers of evaluated lymph nodes), those cases will eventually miss their adequate treatment due to improved lymph node dissection [14]. It does not seem unreasonable to adjust cut-off values that set a minimum number of examined lymph nodes to patient- and tumour-specific parameters that are known to influence the number of lymph nodes [13].

This study is based on a retrospective evaluation and analysis of H&E-stained lymph node slides and clinicopathological data. Regarding size measurement accuracy an interobserver correlation analysis with two independent examiners performed on a sub-cohort of 95 lymph nodes resulted in a high level of concordance (5.76 ± 2,93 mm vs. 5.82 ± 3.00 mm; linear regression: R = 0.992, p < 0.001). Distribution to a different size category occurred in 6 out of 95 cases (6.3%). Not only nodal sizes but also intra-nodal tumour portions were determined and thus allowed calculation of further lymph node features, such as lymph node metastasis to lymph node size ratio (MSR). This was a clear advantage of the study. We want also to mention the disadvantage of different mean numbers of evaluated nodes. Cases from two periods were examined (2002–2004 and 2007–2013). In the former, only conventional lymph node dissection methods were used, in the latter only advanced dissection techniques. This resulted in different mean numbers of evaluated nodes per case between these two periods (2002–2004 vs. 2007–2013: 15.5 vs. 39.4). However, this imbalance was distributed over both study and control group, eliminating statistical bias. The mean number of metastasised lymph nodes between the two periods did not differ significantly (2002–2004 vs. 2007–2013: 3.95 ± 4.05 vs. 4.57 ± 7.30, p = 0.706). Besides that the number of LN5 (>5mm) did not differ significantly either (2002–2004 vs. 2007–2013: 6.78 ± 4.39 bzw. 8.39 ± 6.46 LN5-LK, p = 0.206).

We can assume that a considerable number of metastasised lymph nodes measures less than 6 mm in size; small lymph node metastases contribute to a precise N sub-classification rather than identify nodal positivity as such. Even in node-positive colon cancer cases, enlarged lymph nodes are associated with better survival. Often they are not metastasised but part of a strong immune response.
